# Posttraumatic Parotid Fistula Treated with Transdermal Scopolamine: A Case Report

**DOI:** 10.1155/2012/713148

**Published:** 2012-08-05

**Authors:** Giulio Pagliuca, Salvatore Martellucci, Chiara Rosato, Camilla Gallipoli, Andrea Gallo

**Affiliations:** ENT Section, Department of Surgical Biotechnologies and Science, Sapienza University of Rome, 00185 Rome, Italy

## Abstract

A parotid fistula is a rare and extremely unpleasant condition. In this paper, we present the case of a 53-year-old woman with a diagnosis of posttraumatic fistula of the parotid gland. After exclusion of other therapeutic alternatives, it was decided to use transdermal scopolamine patches at sustained release (Scopoderm TTS). This technique consists in the application every three days of a patch with 1.5 mg of scopolamine in the area of the mastoid apophysis; the patch releases a dose of 0.5 mg of the active substance over each 24-hour period. The patient underwent periodic clinical followup over a period of three years, achieving satisfactory results with no significant adverse effects.

## 1. Introduction

Salivary fistula is a chronic communication between the salivary gland or duct and the skin through which saliva is discharged. Fistulas of the parotid gland are uncommon and result from either ductal or parenchymal injury. The most frequent aetiologies are postoperative complication after parotid gland surgery and accidental trauma [1]. Early detection of injury and prompt treatment are important since fistulas may cause discomfort as well as wound dehiscence and infection [2]. Numerous methods including pressure dressing, total parotidectomy, tympanic neurectomy, radiotherapy, and pharmacotherapy have been advocated in the treatment of salivary fistulas, but none of these has proven to be totally satisfactory [2]. In recent years botulinum toxin has been proposed as primary method for the management of parotid fistula in several reports with encouraging results [3–5]. The use of transdermal scopolamine has recently been described in the management of postrhytidectomtomy parotid fistulas [6]. We report the case of a 53-year-old woman with a parotid fistula secondary to penetrating trauma case of successfully treated using transdermal scopolamine.

## 2. Case Report

**Figure 1:**
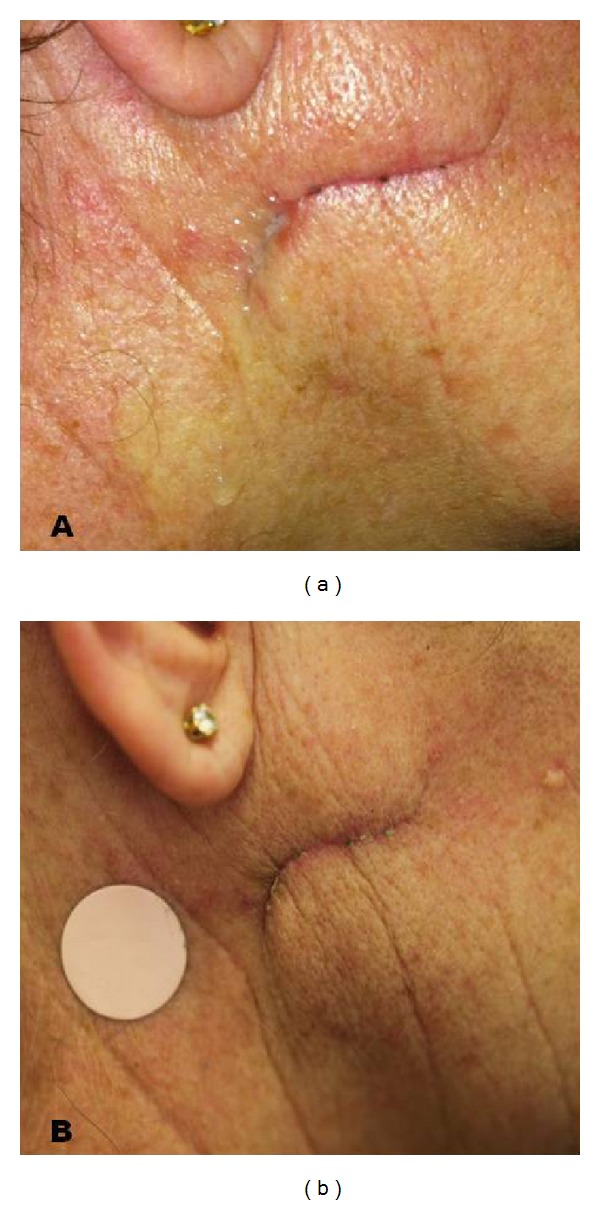
(a) Posttraumatic fistula of right parotid gland. (b) Detail of the application of the scopolamine skin patch.

A 53-year-old woman was taken to the emergency room after an accidental fall. At the moment of the fall, the patient was holding a glass bottle that shattered with the impact. A sharp piece of glass caused a deep wound with a length of about 3 centimetres on the right cheeks. The patient showed no signs of facial nerve injury. The wound was then toileted and sutured. After 10 days from trauma the patient began to complain of the appearance of a continuous colourless and odourless fluid from the wound, which increased after food intake. Therefore the patient came under our observation on 20th day from trauma. A clinical examination revealed on the wound scar a 3 mm orifice discharging a clear serous secretion, suggesting the diagnose of posttraumatic parotid fistula. The patients were invited to limit oral intake for a week in order to reduce salivary output; steristrips were placed to close the fistula and a compressive dressing was applied for 7 days, with no results. Due to severe discomfort caused by a large amount of secretion, a device for transdermal delivery of scopolamine (Transcop Recordati OTC S.p.A. Milan, Italy) was applied on the right preauricular region (Figure 1). This device is a patch of circular form containing a reservoir of 1.5 mg of drug scopolamine, which is released into the bloodstream. The daily release of scopolamine is about 0.5 mg with a duration of a pharmacological three days for each patch. In the following 3 days, the patient reported significant reduction in salivary discharge from the fistula. The patches were replaced and the successive examination after 3 days revealed that the fistula was completely healed. The treatment was well tolerated and the patient referred only to a mild dryness of the mouth in the last 2 days of treatment. No recurrence was observed during a 3-month followup.

## 3. Discussion

Posttraumatic salivary fistulas are often consequent to face's trauma in the course of road accidents where penetrating injuries are associated to the presence of broken glasses [1, 7]. The formation of a salivary fistula after the lesion of glandular parenchyma following a penetrating injury can occur early or late in relation to traumatic event [1, 7]. Although there is consensus in the literature that acute parotid injury must be explored primarily and all injured structures be accurately repaired, in many cases the salivary fistula is not recognized at time of admission because of the coexisting bleeding skin wound. The pathophysiology of healing of parotid fistulas has been well described by Arulpragasam [8]. The growth of granulating tissue heals the leaking acini and ducts. The secretion of saliva, especially during meals, counteracts this healing process. The fistulous opening usually becomes coated with epithelium, and this further contributes to preventing fistula closure. Therefore, when the amount of saliva secretion is suppressed or stopped, healing tends to occur more easily. For this reason therapeutic approaches that aim to depress salivary production by reducing oral intake or using antisialogogues are recommended as first line treatment [1–4]. However, undesirable side effects associated with high peak serum levels following oral or parenteral administration of anticholinergics (such as drowsiness, blurred vision, sedation, confusion, nausea and vomiting, urinary retention, and xerostomia) have precluded their utilization [9–11]. Transcop, developed for the prevention of motion sickness, is a transdermal therapeutic system for controlled delivery for scopolamine. Scopolamine is an antimuscarinic agent that has a powerful action on salivary glands and can reduce the volume of salivary secretion in human beings [12]. The availability of scopolamine in a transdermal delivery system provides an alternative method in the administration of antisialogogues and avoids collateral effects [12, 13]. Absorption occurs at a constant rate to maintain a steady plasma level for at least 72 hours when the patch is applied to the hairless skin behind the ear. It is possible that the application of the circular patch in this region may have a direct effect on the parotid tissue, thus causing a reduction in salivary secretion. The use of transdermal scopolamine to achieve an antisialogogue action has already been described in the management of drooling in patients with neurological disease [12, 13]. Our experience suggests that the use of transdermal scopolamine as antisialogogue can be a conservative, cost effective, and safe strategy for the treatment of posttraumatic parotid fistulas.
